# An In Vitro Evaluation of a New Approach in AMD: Effects of the Combination of Resveratrol and Anti-VEGFs on ARPE-19 Cells

**DOI:** 10.3390/biom16060883

**Published:** 2026-06-15

**Authors:** Onur Konukcu, Mehmet Argun, Semra Acer, Ömer Çelik, Özlem Tök, Levent Tök, Mustafa Nazıroğlu

**Affiliations:** 1 Department of Ophthalmology, Süleyman Demirel University Research and Education Hospital, Isparta TR-32260, Türkiye; 2 Department of Biophysics, Faculty of Medical, Suleyman Demirel University, Isparta TR-32260, Türkiye; 3 Neuroscience Research Center, Suleyman Demirel University, Isparta TR-32260, Türkiye; 4 Drug Discovery Unit, BSN Health, Analyses, Innovation, Consultancy, Organization, Agriculture and Industry Ltd., Göller Bölgesi Teknokenti, Isparta TR-32260, Türkiye

**Keywords:** aflibercept, AMD, ranibizumab, resveratrol, ziv-aflibercept

## Abstract

Anti-vascular endothelial growth factor agents (anti-VEGFs) are the cornerstone of treatment for neovascular age-related macular degeneration (AMD). Resveratrol is a natural polyphenol with well-established antioxidant and anti-apoptotic properties. This study investigated whether resveratrol exerts cytoprotective effects when combined with anti-VEGFs on ARPE-19 cells in vitro. Cells were treated with ranibizumab (RNZ), aflibercept (AFL), or ziv-aflibercept (ZFL), either alone or in combination with resveratrol. Mitochondrial and cytosolic reactive oxygen species (MitROS and CytROS), mitochondrial membrane depolarization (MitDep), caspase-3, -8 and -9 activities, cell viability, apoptosis, and VEGF-A levels were evaluated using confocal microscopy, plate reader-based assays, and ELISA techniques. Anti-VEGFs induced tolerable oxidative or apoptotic stress in ARPE-19 cells but did not exhibit intrinsic antioxidant and cytoprotective effects. The addition of resveratrol significantly enhanced beneficial effects by reducing oxidative stress, preserving mitochondrial integrity, and suppressing intrinsic apoptotic signalling, while increasing cell viability. VEGF-A levels were effectively reduced by anti-VEGF treatment, and this suppression was further augmented by resveratrol without compromising cellular survival. These findings indicate that resveratrol acts as an additive modulator that strengthens the cellular effects of anti-VEGFs on ARPE-19 cells. The combination strategy may represent a supportive approach to optimize long-term anti-VEGF therapy in AMD.

## 1. Introduction

Age-related macular degeneration (AMD) is a chronic and progressive retinal disease that leads to irreversible central vision loss in elderly individuals [1]. AMD represents the most common cause of blindness in developed countries among individuals over 60 years of age and accounts for approximately 8.7% of global blindness, with prevalence expected to increase substantially in the coming decades [2]. The disease is characterized by degeneration of the retinal pigment epithelium (RPE), photoreceptors, and choriocapillaris, ultimately resulting in impaired central visual function.

Vascular endothelial growth factor (VEGF) plays a pivotal role in the development and progression of choroidal neovascularization, which is the primary cause of vision loss in neovascular AMD [3]. Intravitreal anti-VEGF agents, including ranibizumab (RNZ) and aflibercept (AFL), have therefore become the cornerstone of AMD therapy [4]. Ziv-aflibercept (ZFL), although not originally approved for intraocular use, has been increasingly employed in clinical practice due to its cost-effectiveness and comparable efficacy [5].

The RPE is essential for retinal homeostasis, supporting photoreceptor survival through nutrient transport, phagocytosis of photoreceptor outer segments, regulation of oxidative stress, and maintenance of the outer blood–retina barrier. Progressive RPE dysfunction and loss are closely associated with visual decline in both atrophic and neovascular forms of AMD. Oxidative stress and mitochondrial dysfunction have been identified as key contributors to RPE degeneration, leading to activation of apoptotic pathways and cellular loss [6,7].

Based on the pharmacokinetics of ranibizumab during clinical studies, intravitreal administration of ranibizumab (0.5 mg) has an aqueous elimination half-life of about 7.2 days [8]. Meanwhile, aflibercept (2 mg) has been found to have a median aqueous half-life of roughly 11 days, according to human sampling studies [9]. It must be noted, however, that the above half-lives refer only to the rate of elimination from the eyes, while the actual duration of drug effectiveness is also related to additional parameters, such as pharmacodynamics. Ziv-aflibercept possesses the same VEGF-binding fusion proteins as aflibercept but is different in terms of packaging. Repackaging off-label studies showed that ziv-aflibercept retains sterility and VEGF-binding activity if re-packed aseptically into syringes and stored at 4 °C for up to 60 days [10].

Resveratrol (3,5,4′-trihydroxystilbene) is a naturally occurring polyphenolic compound with well-documented antioxidant, anti-inflammatory, anti-apoptotic, and antiangiogenic properties [11,12,13,14]. Previous in vitro studies have demonstrated that resveratrol protects RPE cells against oxidative stress-induced damage and apoptosis, enhances mitochondrial function, and modulates VEGF secretion under hypoxic and inflammatory conditions [15,16]. These properties suggest that resveratrol may act as a supportive agent in therapeutic strategies targeting VEGF signalling. Since resveratrol has incredibly low bioavailability evidence, the current study verifies the paradigm wherein exosomes serve as efficient vectors for delivering drugs to the eye. The potential of exosomes to deliver drug cargo across ocular barriers and the biocompatibility properties of exosomes together help establish the framework of this research work [17].

The present study was designed to investigate whether clinically used anti-VEGF agents exert intrinsic cytoprotective and antioxidative effects on ARPE-19 cells and to determine whether resveratrol enhances these effects in an additive manner. To address this aim, we evaluated mitochondrial and cytosolic reactive oxygen species (MitROS and CytROS) generation, mitochondrial membrane depolarization (MitDep), apoptotic signalling pathways, cell viability, apoptosis, and VEGF-A levels in ARPE-19 cells treated with ranibizumab, aflibercept, or ziv-aflibercept, either alone or in combination with resveratrol.

## 2. Materials and Methods

### 2.1. Chemicals and Reagents

The ARPE-19 cell line was obtained from the American Type Culture Collection (ATCC, Manassas, VA, USA, ATCC Number: CRL-2302). Cell culture media and supplements were purchased from Sigma-Aldrich (Saint Louis, MO, USA). The APOPercentage™ Apoptosis Assay Kit was obtained from Biocolor Ltd. (A1000, Biocolor Ltd., County Antrim, UK). Human VEGF-A ELISA kits were purchased from Bioassay Technology Laboratory (Shanghai, China; Cat. No. E0050Hu). All reagents were of analytical grade. Each experiment was performed at least six times (biological replicates).

### 2.2. Cell Culture

ARPE-19 cells were cultured in a 1:1 mixture of Dulbecco’s Modified Eagle Medium (DMEM) and Ham’s F12 medium supplemented with 10% fetal bovine serum (Biochrom, Berlin, Germany) and 1% penicillin–streptomycin solution (Biochrom, Berlin, Germany), according to the manufacturer’s instructions. Cells were maintained at 37 °C in a humidified incubator with 5% CO_2_ (HF90, Heal Force, Shanghai, China) and used between passages 2 and 12 [18].

### 2.3. Experimental Design

Cells were seeded in appropriate culture plates and allowed to reach suitable confluence prior to treatment. The final volume of culture medium in each experimental condition was set to 4 mL, corresponding to the physiological vitreous volume. Anti-VEGF agents were applied at clinically relevant doses as previously described by Malik et al. [19]. Resveratrol was dissolved in DMSO until the last concentration was less than 0.1% [20]. The concentration of resveratrol (100 µM) was selected based on previous in vitro studies demonstrating cytoprotective and antioxidative effects in ARPE-19 cells without inducing cytotoxicity [21].

The experimental groups were defined as follows:

- Control: ARPE-19 cells incubated for 48 h in standard growth medium.
- Resveratrol (RSV): Cells treated with resveratrol (100 µM) for 48 h.
- Ranibizumab (RNZ): Cells treated with ranibizumab (125 µg/mL) for 48 h [19].
- Aflibercept (AFL): Cells treated with aflibercept (500 µg/mL) for 48 h [19].
- Ziv-aflibercept (ZFL): Cells treated with ziv-aflibercept (500 µg/mL) for 48 h [19].
- RNZ + RSV: Cells treated with ranibizumab (125 µg/mL) and resveratrol (100 µM) for 48 h.
- AFL + RSV: Cells treated with aflibercept (500 µg/mL) and resveratrol (100 µM) for 48 h.
- ZFL + RSV: Cells treated with ziv-aflibercept (500 µg/mL) and resveratrol (100 µM) for 48 h.

### 2.4. Confocal Microscopy Analysis

#### 2.4.1. MitROS, MitDep and CytROS

MitROS, CytROS and MitDep were analyzed using a laser scanning confocal microscope (Zeiss, Oberkochen, Germany) equipped with a Plan-Apochromat 20×/0.8 objective.

MitROS and CytROS were assessed using MitoSOX Red (Thermo Fisher Scientific, Istanbul, Türkiye) (1 µM, 30 min) and DCFH-DA (Sigma Aldrich, St. Louis, MO, USA) (1 µM, 30 min), respectively. MitDep was evaluated using the JC-1 (Santa Cruz, Dallas, TX, USA) fluorescent probe (2 µM, 30 min). Fluorescence intensities were quantified as arbitrary units using ZEN software (Blue edition 3.2, Zeiss, Oberkochen, Germany) [22,23].

#### 2.4.2. Total Number of Cells and Rate of Cell Death

Total cell number and cell death rates were evaluated using Hoechst 33342 (Cell Signaling Technology, Danvers, MA, USA) and propidium iodide (PI) staining. Cells were incubated with Hoechst 33342 (8.1 µM) and PI (Thermo Fisher Scientific, MA, USA) (1.5 µM) for 20 min, followed by confocal imaging. PI-positive cells were counted and expressed as a percentage of total cells using ZEN software [24].

### 2.5. Plate Reader-Based Assays

#### 2.5.1. MitDep and CytROS

MitDep and CytROS levels were also quantified using an automatic microplate reader (Infinite 200 PRO, Tecan Inc., Männedorf, Switzerland) with JC-1 and dihydrorhodamine-123 (DHR-123) (Sigma Aldrich, St. Louis, MO, USA) probes, respectively. Results were normalized to protein content and expressed as a percentage of control values [25,26].

#### 2.5.2. Caspase-3, -8 and -9

Caspase-3, caspase-8, and caspase-9 activities were measured using the fluorogenic substrates Ac-DEVD-AMC, Ac-IETD-AFC, and Ac-LEHD-AFC (Bachem AG, Bubendorf, Switzerland). Fluorescence signals were detected using the microplate reader and expressed as percentages of control values [27].

#### 2.5.3. Cell Viability and Apoptosis

Cell viability was assessed using the MTT assay. After treatment, cells were incubated with MTT reagent for 90 min at 37 °C, followed by solubilization of formazan crystals with DMSO. Absorbance was measured at 490 nm with a reference wavelength of 650 nm, and results were expressed as fold change relative to the control [27,28].

Apoptosis was quantified using the APOPercentage™ Apoptosis Assay Kit according to the manufacturer’s protocol. Results were expressed as percentages of control values [25,29].

### 2.6. VEGF-A Measurement

VEGF-A concentrations in culture supernatants were measured using a commercial ELISA kit (Bioassay Technology Laboratory, Zhejiang, China) according to the manufacturer’s instructions. The standard curve demonstrated a coefficient of determination (R^2^) of 0.998, and results were expressed in pg/mL [18,21].

### 2.7. Statistical Analysis

All data were expressed as mean ± standard deviation. Statistical analyses were performed using one-way analysis of variance (ANOVA) followed by Tukey’s post hoc test for multiple comparisons (GraphPad Prism v8.0, GraphPad Software, CA, USA). A *p*-value < 0.05 was considered statistically significant.

## 3. Results

**Figure 1:**
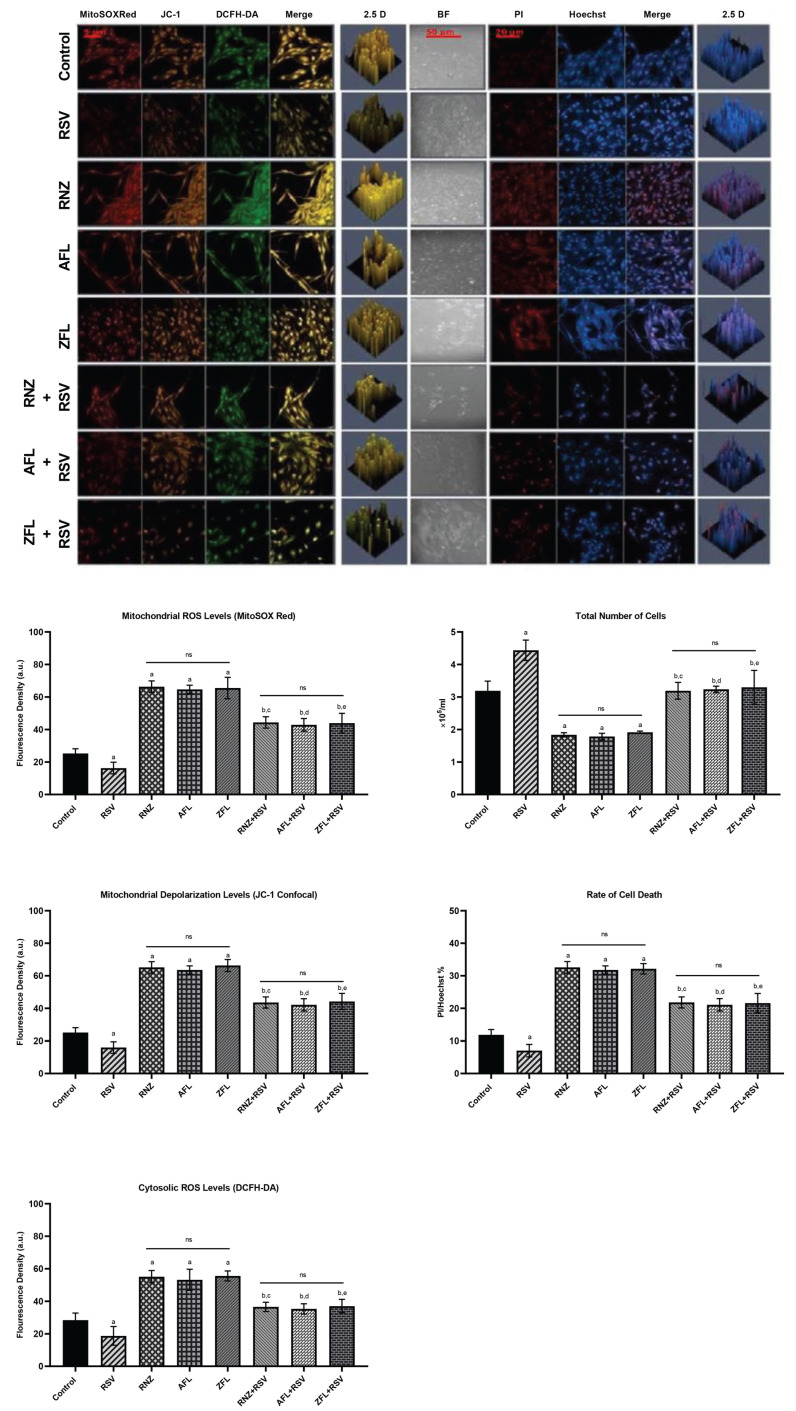
Confocal microscopy images of the experimental groups (mean ± SD; *n* = 6).

The confocal microscopy images are shown in Figure 1.

### 3.1. Effects of Anti-VEGF Agents and Resveratrol on MitROS and CytROS Production

**Figure 2:**
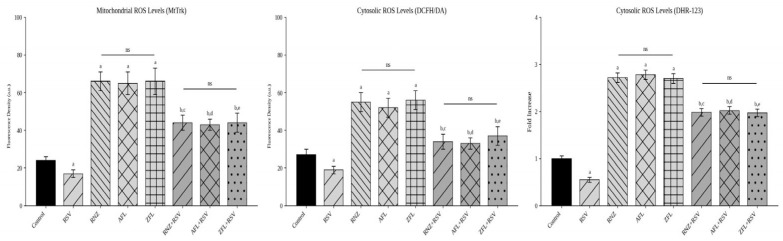
Mitochondrial reactive oxygen species (MitROS) and Cytosolic reactive oxygen species (CytROS) levels (mean ± SD; *n* = 6) (^a^ *p* < 0.001 vs. Control group, ^b^ *p* < 0.001 vs. resveratrol (RSV) group, ^c^ *p* < 0.001 vs. ranibizumab (RNZ) group, ^d^ *p* < 0.001 vs. aflibercept (AFL) group, ^e^ *p* < 0.001 vs. ziv-aflibercept (ZFL) group, ns: not significant).

MitROS and CytROS levels were evaluated in ARPE-19 cells following treatment with anti-VEGF agents and resveratrol, either alone or in combination. Compared with the control group, treatment alone with ranibizumab, aflibercept, or ziv-aflibercept induced an increase in MitROS or CytROS levels (*p* < 0.001). Resveratrol treatment alone significantly decreased MitROS and CytROS levels compared with control cells (*p* < 0.001). The combination of resveratrol with each anti-VEGF agent resulted in a reduction in ROS levels compared with treatments alone anti-VEGFs (*p* < 0.001). Confocal microscopy findings were consistent with plate reader-based quantitative analyses, confirming the reliability of the observed ROS modulation across experimental platforms (Figure 2).

### 3.2. Anti-VEGF Agents Preserve Mitochondrial Membrane Integrity and Are Potentiated by Resveratrol

MitDep was assessed using JC-1 fluorescence analysis. None of the anti-VEGF agents caused MitDep when compared with control cells. Instead, ranibizumab, aflibercept, and ziv-aflibercept significantly preserved MitDep (*p* < 0.001).

**Figure 3:**
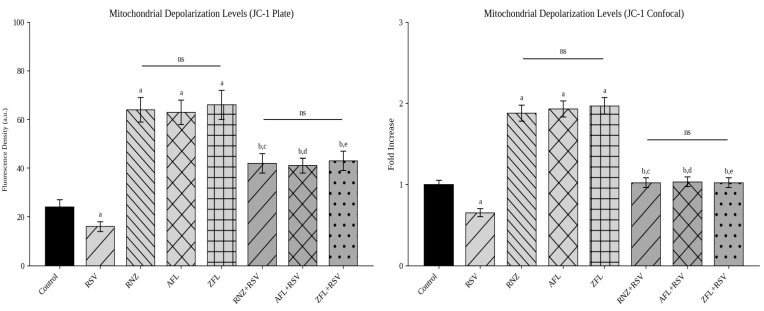
Mitochondrial membrane depolarization (MitDep) levels (mean ± SD; *n* = 6) (^a^ *p* < 0.001 vs. Control group, ^b^ *p* < 0.001 vs. RSV group, ^c^ *p* < 0.001 vs. RNZ group, ^d^ *p* < 0.001 vs. AFL group, ^e^ *p* < 0.001 vs. ZFL group, ns: not significant).

Resveratrol alone exerted a strong protective effect against MitDep (*p* < 0.001). Combined treatment with resveratrol and anti-VEGF agents further enhanced mitochondrial membrane stability, resulting in significantly lower MitDep values compared with either anti-VEGF or resveratrol monotherapy (*p* < 0.001) (Figure 3).

### 3.3. Modulation of Apoptotic Signalling Pathways

Caspase-3, caspase-8, and caspase-9 activities were measured to evaluate apoptotic signalling. Treatment with anti-VEGF agents alone did not increase the activity of any caspase compared with controls. Instead, a significant reduction in caspase-3 and caspase-9 activities was observed in all anti-VEGF-treated groups (*p* < 0.001), whereas caspase-8 activity remained unchanged.

**Figure 4:**
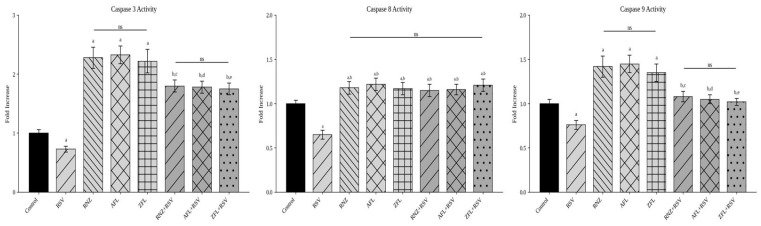
Caspase-3, caspase-8 and caspase-9 activity levels by plate reader (mean ± SD; *n* = 6) (for caspase-3 and caspase-9; ^a^ *p* < 0.001 vs. Control group, ^b^ *p* < 0.001 vs. RSV group, ^c^ *p* < 0.001 vs. RNZ group, ^d^ *p* < 0.001 vs. AFL group, ^e^ *p* < 0.001 vs. ZFL group) (for caspase-8; ^a^ *p* < 0.001 vs. Control group, ^b^ *p* < 0.001 vs. RSV group, ns: not significant).

Resveratrol treatment alone significantly suppressed caspase-3 and caspase-9 activities (*p* < 0.01). Importantly, combination therapy with resveratrol and anti-VEGF agents resulted in a more pronounced suppression of intrinsic apoptotic signalling, as reflected by further reductions in caspase-3 and caspase-9 activities compared with monotherapy groups (*p* < 0.001) (Figure 4).

### 3.4. Effects on Cell Viability and Apoptosis

**Figure 5:**
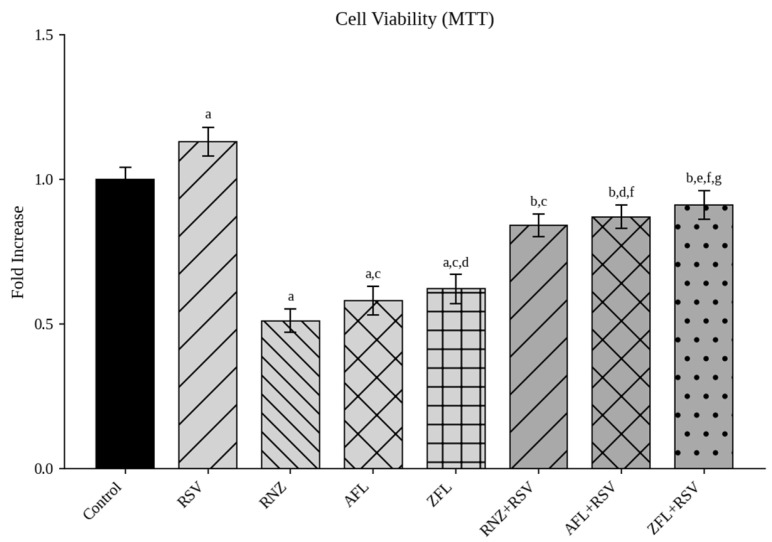
Cell viability levels by plate reader (mean ± SD; *n* = 6) (^a^ *p* < 0.001 vs. Control group, ^b^ *p* < 0.001 vs. RSV group, ^c^ *p* < 0.001 vs. RNZ group, ^d^ *p* < 0.001 vs. AFL group, ^e^ *p* < 0.001 vs. ZFL group, ^f^ *p* < 0.001 vs. RNZ + RSV group, ^g^ *p* < 0.001 vs. AFL + RSV group).

Cell viability was assessed using the MTT assay. Treatment with anti-VEGF agents alone reduced ARPE-19 cell viability compared with control cells, but not combined groups. Resveratrol alone significantly enhanced cell viability compared with the control (*p* < 0.001). Combined treatment anti-VEGFs with resveratrol lead an increase in cellular viability compared to monotherapy anti-VEGF treatment (*p* < 0.001) (Figure 5).

**Figure 6:**
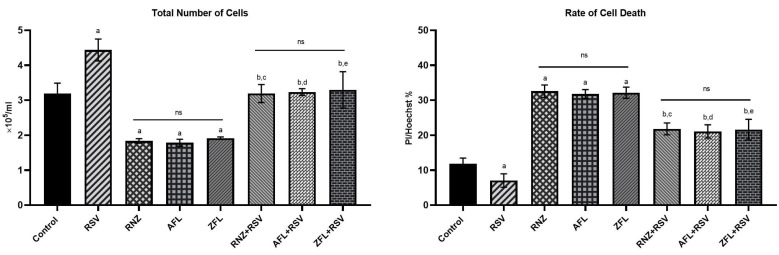
Total number of cells and rate of cell death (mean ± SD; *n* = 6) (^a^ *p* < 0.001 vs. Control group, ^b^ *p* < 0.001 vs. RSV group, ^c^ *p* < 0.001 vs. RNZ group, ^d^ *p* < 0.001 vs. AFL group, ^e^ *p* < 0.001 vs. ZFL group, ns: not significant).

The total number of dead cells and the rate of cell death were evaluated in ARPE-19 cultures. The total number of cells in sole anti-VEGF-treated groups significantly decreased compared to the control group (*p* < 0.001), while no change was observed in combined groups compared to the control group (*p* > 0.001). Treatment with ranibizumab, aflibercept, or ziv-aflibercept alone resulted in a significant increase in the total number of dead cells compared with the control group (*p* < 0.001). Treatment with resveratrol alone significantly reduced the rate of cell death and caused an increase in the total number of cells compared with control cells (*p* < 0.001). Compared to the control group, the use of anti-VEGFs alone resulted in an increase in cell death (*p* < 0.001), while no significant increase in cell death was observed when anti-VEGFs were used in combination with resveratrol (*p* > 0.001) (Figure 6).

**Figure 7:**
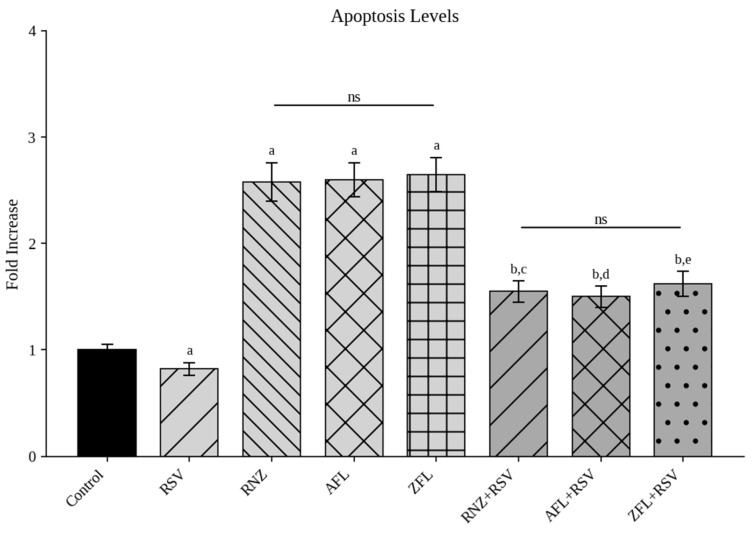
Apoptosis levels by plate reader (mean ± SD; *n* = 6) (^a^ *p* < 0.001 vs. Control group, ^b^ *p* < 0.001 vs. RSV group, ^c^ *p* < 0.001 vs. RNZ group, ^d^ *p* < 0.001 vs. AFL group, ^e^ *p* < 0.001 vs. ZFL group, ns: not significant).

Analysis using the APOPercentage™ assay revealed a significant increase in apoptotic cell death in only anti-VEGF-treated groups. According to MTT results, there was no statistical significance between the control and combined resveratrol groups (RNZ + RSV, AFL + RSV and ZFL + RSV), although a significant difference was determined between the control and only anti-VEGF groups (RNZ, AFL and ZFL) (*p* < 0.001) (Figure 7).

### 3.5. Regulation of VEGF-A Levels

VEGF-A concentrations in cell culture supernatants were measured by ELISA. Treatment with ranibizumab, aflibercept, or ziv-aflibercept resulted in a significant reduction in VEGF-A levels compared with control cells (*p* < 0.001). Resveratrol alone also caused a moderate decrease in VEGF-A levels (*p* < 0.01).

**Figure 8:**
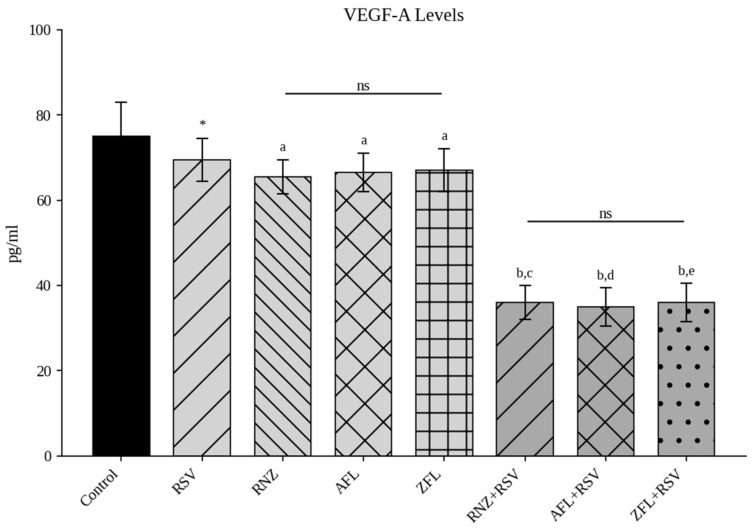
Vascular endothelial growth factor-A (VEGF-A) levels by plate reader (mean ± SD; *n* = 6) (* *p* < 0.01 vs. Control group, ^a^ *p* < 0.001 vs. Control group, ^b^ *p* < 0.001 vs. RSV group, ^c^ *p* < 0.001 vs. RNZ group, ^d^ *p* < 0.001 vs. AFL group, ^e^ *p* < 0.001 vs. ZFL group, ns: not significant).

Notably, combination treatment with resveratrol and anti-VEGF agents led to a further reduction in VEGF-A levels compared with anti-VEGF monotherapy (*p* < 0.001), without compromising cell viability or inducing apoptotic stress (Figure 8).

## 4. Discussion

The present study demonstrates that clinically used anti-vascular endothelial growth factor (anti-VEGF) agents may induce moderate oxidative or apoptotic stress in ARPE-19 cells. It may be hypothesized that the accumulation of intravitreal anti-VEGF agents leads to oxidative stress within the cell activities, eventually leading to the dysfunction of the retinal pigment epithelium. Combining the anti-VEGFs with resveratrol caused a reduction in oxidative stress, improved mitochondrial depolarization, suppressed intrinsic apoptotic signalling, and increased cellular viability. These findings provide important insights into the cellular effects of anti-VEGF therapy on retinal pigment epithelium (RPE) cells, and support the potential role of resveratrol as a supportive modulator.

VEGF plays a central role in retinal physiology and pathology, particularly in the development of choroidal neovascularization associated with neovascular AMD. Anti-VEGF agents such as ranibizumab and aflibercept have become the cornerstone of AMD treatment due to their efficacy in suppressing pathological angiogenesis and stabilizing vision. Although concerns have occasionally been raised regarding potential adverse cellular effects of long-term VEGF suppression, accumulating experimental and clinical evidence supports the overall safety of these agents on retinal cells [19,30,31,32,33,34,35,36,37,38,39,40,41]. When establishing the anti-VEGF dose for the cell culture experiment, it was assumed that the volume of vitreous humour is around 4 mL, and hence the appropriate dose of anti-VEGF was provided accordingly [19].

In the present study, the combination of resveratrol with anti-VEGF agents decreased MitROS or CytROS production, MitDep, caspase activation, and apoptotic cell death. In this study, cell culture supernatants were utilized for measuring VEGF-A levels secreted by ARPE-19 cells, like many studies in the literature [42], but it does not mean that its extracellular quantity reflects the VEGF-A production in the cell.

The RPE is highly susceptible to oxidative damage due to its high metabolic demand and continuous exposure to light and oxygen [43,44]. Preservation of mitochondrial function and prevention of apoptosis in RPE cells are therefore critical for maintaining retinal homeostasis and long-term visual function. The observed antioxidative and cytoprotective effects of combined resveratrol anti-VEGF agents in this study support their favourable safety profile and suggest that their benefits may extend beyond angiogenesis inhibition.

Resveratrol is a well-characterized polyphenolic compound with potent antioxidant, anti-inflammatory, and anti-apoptotic properties. Numerous in vitro studies have demonstrated that resveratrol protects cells from oxidative stress-induced damage, preserves mitochondrial function, and suppresses intrinsic apoptotic pathways [12,13,14]. In line with these findings, resveratrol alone significantly reduced oxidative stress, stabilized mitochondrial membrane potential, decreased caspase-3 and caspase-9 activities, and enhanced cell viability in ARPE-19 cells.

It has been documented that two different pathways are responsible for cell apoptosis: the intrinsic pathway and the extrinsic pathway. Even though there is a difference in their upstream signalling, their common target is the activation of executioner caspases, caspase-3, and caspase-7 [45]. Levels of caspase-8 (extrinsic pathway) were not meaningfully reduced in all groups except the resveratrol group, compared to the control group. It was concluded that raised activity of caspase-3 and caspase-7 triggered apoptosis intrinsically (mitochondrial pathway) in our experiment.

Importantly, the combined treatment of resveratrol with anti-VEGF agents resulted in protective effects across all evaluated parameters. The combination further reduced oxidative stress, enhanced mitochondrial stability, suppressed intrinsic apoptotic signalling, and increased cell viability compared with anti-VEGF monotherapy. Notably, this enhancement occurred without compromising the VEGF-A-suppressive efficacy of anti-VEGF agents, indicating that resveratrol does not interfere with the primary therapeutic mechanism of VEGF inhibition.

Similarly, VEGF-A levels were significantly reduced in all anti-VEGF-treated groups, and the addition of resveratrol resulted in a further decrease without negatively affecting cell viability. This finding suggests that resveratrol may reinforce the antiangiogenic efficacy of anti-VEGF therapy while simultaneously supporting RPE cell health, a relevant aspect in the long-term management of AMD.

The additive nature of resveratrol’s effects suggests that it may act as a supportive modulator that reduces the limited oxidative cellular damage created by anti-VEGF therapy. This interaction may be particularly relevant in the context of long-term treatment, where preservation of RPE integrity is essential for sustained therapeutic success. By reinforcing antioxidative defences and mitochondrial function, resveratrol may contribute to improved cellular resilience during chronic anti-VEGF exposure.

The observed cytoprotective effect observed in the combination treatment groups may be attributed to complementary molecular pathways. While anti-VEGF agents modulate VEGF-dependent signalling, resveratrol exerts broader antioxidative and mitochondrial stabilizing actions, including reactive oxygen species scavenging and suppression of intrinsic apoptotic cascades. The convergence of these pathways likely underlies the enhanced preservation of mitochondrial integrity and cell survival observed in our study.

Among the anti-VEGF agents evaluated, ziv-aflibercept demonstrated cytoprotective effects comparable to those of ranibizumab and aflibercept, with slightly higher cell viability observed in some parameters. These findings are consistent with previous reports supporting the safety and efficacy of ziv-aflibercept and further indicate that the additive benefit of resveratrol is not agent-specific but rather reflects a class-wide interaction with anti-VEGF therapy [46,47,48,49,50,51].

This study primarily aimed to investigate functional cellular outcomes rather than detailed molecular signalling pathways. Future research, by evaluating possible signalling mechanisms, may further elucidate the molecular basis of the observed cytoprotective interaction.

Several limitations of this study should be acknowledged. The findings are based on an in vitro ARPE-19 cell model and may not fully replicate the complex in vivo retinal microenvironment. Similarly, the low oral bioavailability of resveratrol may limit its in vivo benefits. While various studies conducted experimentally and cited in the literature have shown that 100 µM resveratrol when administered through intravitreal delivery is able to prevent retinal ganglion cell death after retinal ischemia–reperfusion injury, with 100 µM resveratrol being the optimal concentration for such action, these experiments, including the one conducted by us, do not amount to physiological proof that resveratrol is able to reach such a level (100 µM) in the vitreous [52,53,54]. Endothelial cells and photoreceptors were not included, despite their relevance to AMD pathophysiology. Nevertheless, the use of ARPE-19 cells is appropriate for investigating RPE-specific cellular responses, given the central role of the RPE in retinal homeostasis and disease progression. Additionally, only a single concentration of resveratrol and anti-VEGF agents was evaluated. The experiments were conducted under basal culture conditions rather than in an oxidative stress-induced disease model. A dual loaded nanoparticulate system comprising resveratrol and metformin by intravitreal injection has been found to achieve a 15 times increase in the permeation of retina. Such an approach can prevent exhaustion of intrinsic antioxidant levels and block the abnormal growth of blood vessels within the retina up to 56 days [55]. Further pre-clinal and in vivo experiments are needed to support our findings.

It has been revealed that exosomal microRNAs and proteins are selectively sorted and regulate cell migration, proliferation, wound healing, and expression of stem cell markers [56]. This suggests that small vesicle-mediated cargo delivery is critical for determining phenotypes of ocular cells [56]. Future studies incorporating in vivo models and dose–response analyses are warranted to further elucidate the clinical relevance of these findings.

It was reported that many extracellular stimuli such as hormones, oxidative stress, ultraviolet radiation, and hypoxia can trigger the MAPK pathway, which is an intracellular serine-threonine protein kinase [57,58]. Additionally, it was postulated that the mammalian target of rapamycin (mTOR) may lead to the phagocytosis of RPE cells by acting on nuclear factor erythroid 2-related factor 2 (Nrf-2), acting as an antioxidant protein and transcription factor [57].

## 5. Conclusions

In conclusion, this study demonstrates that anti-VEGF agents combined with resveratrol exert intrinsic antioxidative and cytoprotective effects on ARPE-19 cells in vitro, and do not induce oxidative or apoptotic stress. Resveratrol enhances these beneficial effects in a manner that does not interfere with VEGF-A suppression. These results support the potential of resveratrol as a supportive strategy to optimize the cellular effects of anti-VEGF therapy in the management of neovascular AMD. Further in vivo and clinical studies are warranted to evaluate the translational relevance of this combination strategy in AMD management.

## Data Availability

The raw data supporting the conclusions of this article will be made available by the authors on request.

## Abbreviations

The following abbreviations are used in this manuscript:

AMD	Age-Related Macular Degeneration
VEGF	Vascular Endothelial Growth Factor
RPE	Retina Pigment Epithelium
RSV	Resveratrol
AFL	Aflibercept
ZFL	Ziv-Aflibercept
RNZ	Ranibizumab
MitROS	Mitochondrial reactive oxygen species
CytROS	Cytosolic reactive oxygen species
MitDep	Mitochondrial membrane depolarization
